# Increased Intrathecal Immune Activation in Virally Suppressed HIV-1 Infected Patients with Neurocognitive Impairment

**DOI:** 10.1371/journal.pone.0157160

**Published:** 2016-06-13

**Authors:** Arvid Edén, Thomas D. Marcotte, Robert K. Heaton, Staffan Nilsson, Henrik Zetterberg, Dietmar Fuchs, Donald Franklin, Richard W. Price, Igor Grant, Scott L. Letendre, Magnus Gisslén

**Affiliations:** 1 Department of Infectious Diseases, Institute of Biomedicine, Sahlgrenska Academy, University of Gothenburg, Gothenburg, Sweden; 2 Department of Psychiatry, University of California San Diego, San Diego, California, United States of America; 3 Mathematical Sciences, Chalmers University of Technology, Gothenburg, Sweden; 4 Department of Psychiatry and Neurochemistry, Institute of Neuroscience and Physiology, Sahlgrenska Academy, University of Gothenburg, Gothenburg, Sweden; 5 Institute of Neurology, University College London, London, United Kingdom; 6 Division of Biological Chemistry, Biocenter, Innsbruck Medical University, Innsbruck, Austria; 7 Department of Neurology, University of California San Francisco, San Francisco, California, United States of America; UNSW Australia, AUSTRALIA

## Abstract

**Objective:**

Although milder forms of HIV-associated neurocognitive disorder (HAND) remain prevalent, a correlation to neuronal injury has not been established in patients on antiretroviral therapy (ART). We examined the relationship between mild HAND and CSF neurofilament light protein (NFL), a biomarker of neuronal injury; and CSF neopterin, a biomarker of CNS immunoactivation, in virally suppressed patients on antiretroviral therapy (ART).

**Design and Methods:**

We selected 99 subjects on suppressive ART followed longitudinally from the CNS HIV Anti-Retroviral Therapy Effects Research (CHARTER) study. Based on standardized comprehensive neurocognitive performance (NP) testing, subjects were classified as neurocognitively normal (NCN; n = 29) or impaired (NCI; n = 70). The NCI group included subjects with asymptomatic (ANI; n = 37) or mild (MND; n = 33) HAND. CSF biomarkers were analyzed on two occasions.

**Results:**

Geometric mean CSF neopterin was 25% higher in the NCI group (p = 0.04) and NFL and neopterin were significantly correlated within the NCI group (r = 0.30; p<0.001) but not in the NCN group (r = -0.13; p = 0.3). Additionally, a trend towards higher NFL was seen in the NCI group (p = 0.06).

**Conclusions:**

Mild HAND was associated with increased intrathecal immune activation, and the correlation between neopterin and NFL found in NCI subjects indicates an association between neurocognitive impairment, CNS inflammation and neuronal damage. Together these findings suggest that NCI despite ART may represent an active pathological process within the CNS that needs further characterization in prospective studies.

## Introduction

With antiretroviral therapy (ART) the incidence of HIV-associated dementia (HAD), the most severe form of HIV-associated neurocognitive disorders (HAND), has been dramatically reduced [1, 2]. However, despite this clear effect on more severe CNS disease, a number of studies have reported a substantial prevalence of mainly the milder HAND forms asymptomatic neurocognitive impairment (ANI) and mild neurocognitive disorder (MND) ranging from 18% to as high as 35–69% [2–5]. The formal diagnosis of HAND utilizes neuropsychological testing in comparison to normative controls [3]. However, neuropsychological testing does not distinguish between impairment resulting from ongoing brain injury or from static residual damage due to prior events, a distinction that has important implications for therapeutic strategies. CSF biomarkers that reflect processes within the brain constitute an important complementary approach to evaluate ongoing CNS disease as well as responses to ART [6, 7].

The light subunit of the neurofilament protein (NFL) is a major structural element of myelinated axons, and NFL concentration in CSF is a sensitive marker of neuronal damage in several neurologic diseases[8–10]. In HIV-1 disease, CSF NFL levels are substantially increased in patients with HAD [11, 12], but decreases and are frequently normalized with initiation of ART. CSF neopterin is a marker of macrophage and microglial activation. While ART generally reduces immune activation, many patients still have residual immune activation in the CNS despite viral suppression, and CSF neopterin frequently remains elevated even in patients on long-term effective therapy [13, 14].

Although the clinical relevance of the milder forms of HAND, particularly ANI, has been questioned [15, 16], recent data have suggested an increased risk of progression to symptomatic impairment in subjects diagnosed with ANI compared to neurocognitively unimpaired subjects, supporting the value of an ANI diagnosis in the clinical setting [17]. However, it is not fully established whether or not progression in neurocognitive impairment represents an ongoing pathologic process within the CNS that continues even in the face of functioning ART. Although residual inflammatory activity in the CNS is present in many virally suppressed patients, the clinical significance of continuous low level intrathecal immune activation during effective ART remains unclear. The aims of the current study were to investigate if milder forms of HAND (ANI and MND) were associated with CSF markers of neuronal damage (NFL) and immune activation (neopterin) despite effective viral suppression, and if CSF biomarkers were associated with disease progression in patients with neurocognitive impairment.

## Methods

### Study design and subjects

We identified subjects from longitudinal neuroAIDS studies at the HIV Neurobehavioral Research Program (HNRP), including the CNS HIV Anti-Retroviral Therapy Effects Research (CHARTER) study, an ongoing observational multicenter cohort study which has been described in detail previously [2]. Individual subjects who were selected for inclusion in the current analysis were all on combination antiretroviral therapy with viral suppression in plasma (HIV-1 RNA <50 copies/ml at inclusion), had available stored samples of plasma and CSF, comprehensive neuropsychological assessments, and had no significant comorbidities (e.g., current substance dependence, cerebrovascular disease, severe psychiatric or neurological disorders) that would have a confounding impact on neurocognitive performance or CSF biomarkers. For the purpose of the current analysis, subjects were classified as neurocognitively normal (NCN) or neurocognitively impaired (NCI), based on results from neuropsychological (NP) testing using normative adjustments and assessing seven cognitive domains. Overall cognitive status was determined using the Global Deficit Score (GDS) approach, which gives greater weight to impaired performance, as would be done by a clinician, and has been shown to be sensitive to HIV-related impairments [18, 19]. Change in neurocognitive performance was determined in relation to published normative standards as described previously [20, 21]. The NCI group contained subjects diagnosed with either ANI or MND according to the Frascati criteria [3]. Although MND requires minor interference in daily functioning, subjects with ANI and MND otherwise demonstrate identical degrees of impairment in NP-testing, and were collectively compared to unimpaired subjects in the current analysis. Subjects were examined on two separate occasions to allow evaluation of the NP-stability and change in CSF biomarkers over time, and were selected to yield approximately a 2:1 sample of patients with NCI vs. NCN, with a roughly equal distribution of subjects with ANI or MND within the NCI group. In addition, to evaluate an association between the CSF biomarkers and longitudinal NP decline, subjects with NCI were selected to include individuals with either stable or declining neurocognitive performance.

### Study procedures

Subjects included from the longitudinal studies underwent a venipuncture, lumbar puncture (LP), neurological assessment, comprehensive neurocognitive testing, detailed substance use history, a fully-structured psychiatric interview for lifetime and current (30-day) diagnoses of major depression and alcohol and other psychoactive substance use disorders, and a measure of mood symptoms in the previous fourteen days. Details of the assessments, including assessment of comorbidities, have been reported in detail previously [2, 17].

Data collection for the study was approved by the UCSD Human Research Protection Program (# 110089), and all study participants provided written informed consent. De-identified data and biological samples were obtained with the permission of the CHARTER steering committee and HNRP Research Review Committee.

### Laboratory methods

Clinical laboratory assessments of CSF WBC and CD4+ T-lymphocyte count were performed at each participating site. Plasma and CSF HIV-1 RNA was quantified using RT-PCR (Amplicor® version 1.5, Roche Diagnostic Systems) with a lower limit of quantification of 50 copies/ml.

For CSF biomarker analysis, we used cell-free specimens of CSF stored at -80°C and not thawed until analysis. CSF NFL was measured using a sandwich ELISA method with a lower limit of quantification of 50 ng/l (NF-light® ELISA kit, UmanDiagnostics AB, Umeå, Sweden) at the Clinical Neurochemistry Laboratory at the University of Gothenburg according to the manufacturers description. CSF NFL concentrations have previously been shown to increase with normal ageing in healthy individuals [12]. The upper normal reference limits of CSF NFL previously established using CSF samples from 108 neurologically healthy control individuals were <380 ng/l (18–29 years), <560 (30–39 years), <890 (40–59 years), <1850 (>59 years). CSF neopterin was analyzed using a commercially available immunoassay (BRAHMS, Berlin, Germany), with an upper normal reference value of 5.8 nmol/l [22].

### Statistical analysis

Descriptive statistics were performed using Prism (version 6, Graph-Pad) or SPSS (IBM SPSS version 20) software. Continuous variables were log_10_ transformed where appropriate for the tests used. For comparisons of continuous variables between independent groups, the Mann-Whitney test or *T*-test was used, as appropriate. Comparisons of categorical variables were analyzed using Fishers exact test or χ^2^-test. Correlations were explored using Pearson correlation. Analyses involving repeated measurements were performed with linear mixed effects models.

## Results

### Study population

Ninety-nine subjects (90% male) who fulfilled inclusion criteria were identified from the HNRP studies and included in the analysis. Twenty-nine subjects were classified as NCN and 70 as NCI (37 with ANI and 33 with MND). The median (IQR) interval between visits for the whole study population was 249 (182–366) days. Subject baseline characteristics of the two groups are shown in Table 1.

**Table 1:** 

	Baseline characteristics		
Subject group	NPN (n = 29)	NCI (n = 70)	*p* Value
*GDS*, *median (IQR)*	*0*.*067 (0–0*.*26)*	*0*.*87 (0*.*66–1*.*41)*	-
Age	45 (39–53)	48 (42–55)	
Male sex (%)	26/29 (90%)	64/70 (91%)	
Ethnicity			
White (%)	21 (72%)	49 (70%)	
Hispanic (%)	3 (10%)	7 (10%)	
African American (%)	3 (10%)	11 (16%)	
Nadir CD4, median (IQR) cells/mm3	120 (10–274)	64 (10–206)	
Current CD4, median (IQR) cells/mm3	602 (453–899)	503 (334–685)	0.04
CSF WBC	2 (1–3)	1 (1–2)	
Undetectable in plasma, median (IQR) days	783 (354–1689)	382 (0–1084)	0.02
Undetectable in CSF, median (IQR) days	455 (188–1085)	363 (0–600)	
Lifetime major depression, n (%)	14/26 (54%)	39/66 (59%)	
Current major depression, n (%)	2/25 (8%)	7/65 (11%)	
Time between visits, median (IQR)	329 (177–370)	241 (183–364)	
ART regimen			
NNRTI + NRTIs	12 (41%)	24 (34%)	
PI+/-r + NRTIs	11 (38%)	31 (44%)	
INSTI + NRTIs	0	2 (3%)	
Other*	6 (21%)	13 (19%)	

NPN, neurocognitive performance normal; NCI, neurocognitively impaired; GDS, global deficit score; IQR, interquartile range; CSF, cerebrospinal fluid; WBC, white blood cell count; ART, antiretroviral therapy; NNRTI, non-nucleoside reverse transcriptase inhibitor; NRTI, nucleos(t)ide reverse transcriptase inhibitor; PI, protease inhibitor; INSTI, integrase strand transfer inhibitor.

* including PI+NNRTI+NRTIs; NNRTI+INSTI+NRTIs; PI+INSTI+NRTIs

All subjects were on combination ART with plasma HIV-1 RNA <50 copies/ml at baseline. All but one subject (CSF HIV-1 RNA 52 copies/ml) also had a baseline HIV-1 RNA <50 copies/ml in CSF. ART regimens were similar in the two patient groups (Table 1). By study design, the median (IQR) GDS was higher in the NCI group (0.87 [0.63–1.4]) than in the NCN group (0.067 [0–0.26]). Current CD4^+^ T- cell count was high in both groups, although statistically significantly lower in the NCI subject group at baseline (Table 1), but not at follow up. At follow up, median (IQR) CD4^+^ T-cell count was 514 (349–776) cells/mm^3^ in the NCI group, and 640 (418–941) cells/mm3 in the NCN group (p = 0.12). Additionally, the NCN group had a longer history of viral suppression in plasma than the NCI group (Table 1). Although subjects in the NCI group tended to have a lower nadir CD4^+^ T-cell count than the unimpaired subject group, this difference was not statistically significant. The rate of major depression, either lifetime or current, did not differ in the two subject groups.

At follow up, three patients had detectable HIV-1 RNA in plasma (75, 76 and 184 copies/ml), two in CSF (59 and 925 copies/ml) and one patient had detectable HIV-1 RNA in both plasma and CSF (106 and 74 copies/ml, respectively), all in the NCI group. All subjects with detectable CSF HIV-1 RNA had CSF neopterin above the upper normal reference value at follow up (range 15.1–60 nmol/l). However, none of the subjects with viral RNA above detection level in either plasma or CSF had CSF NFL above the upper normal age-adjusted reference.

### CSF biomarkers and neurocognitive impairment

**Fig 1:**
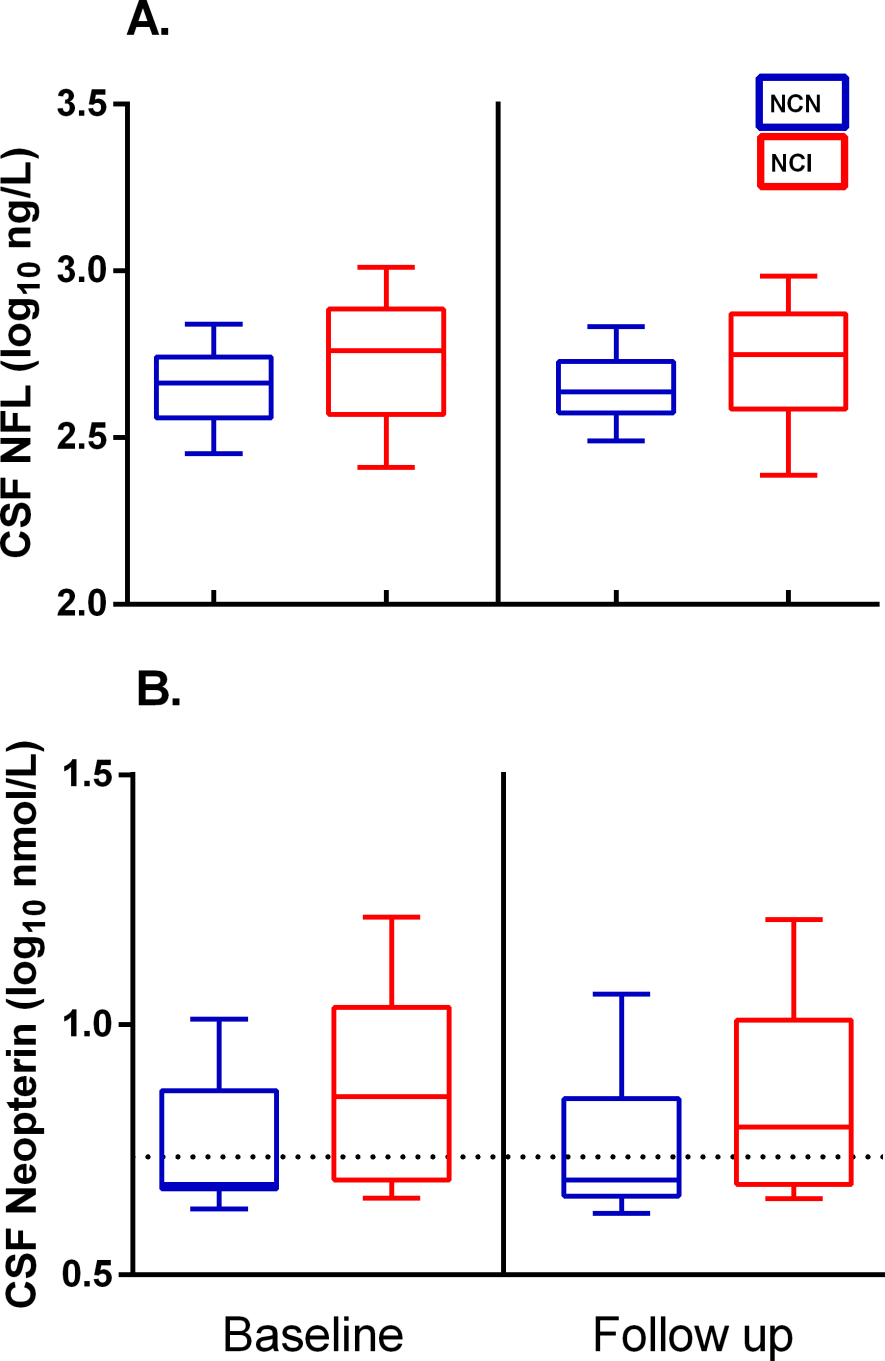
Overview of CSF biomarker distribution in the study groups. Baseline and follow up distribution of CSF NFL (A.) and neopterin (B.) in neurocognitively normal (NCN) and neurocognitively impaired (NCI) subjects. Whiskers show 10-90^th^ percentile. Values are log_10_ transformed for presentation. Dotted line represents upper normal reference for CSF neopterin (5.8 nmol/l).

An overview of the distribution of CSF biomarkers at baseline and follow up in the study groups is shown in Fig 1. Baseline median (IQR) CSF NFL levels were 577 (371–769) ng/l in the NCI group and 462 (364–552) ng/l in the NCN group. At follow up, corresponding levels were 561 (385–746) and 435 (375–535) ng/l, respectively.

**Fig 2:**
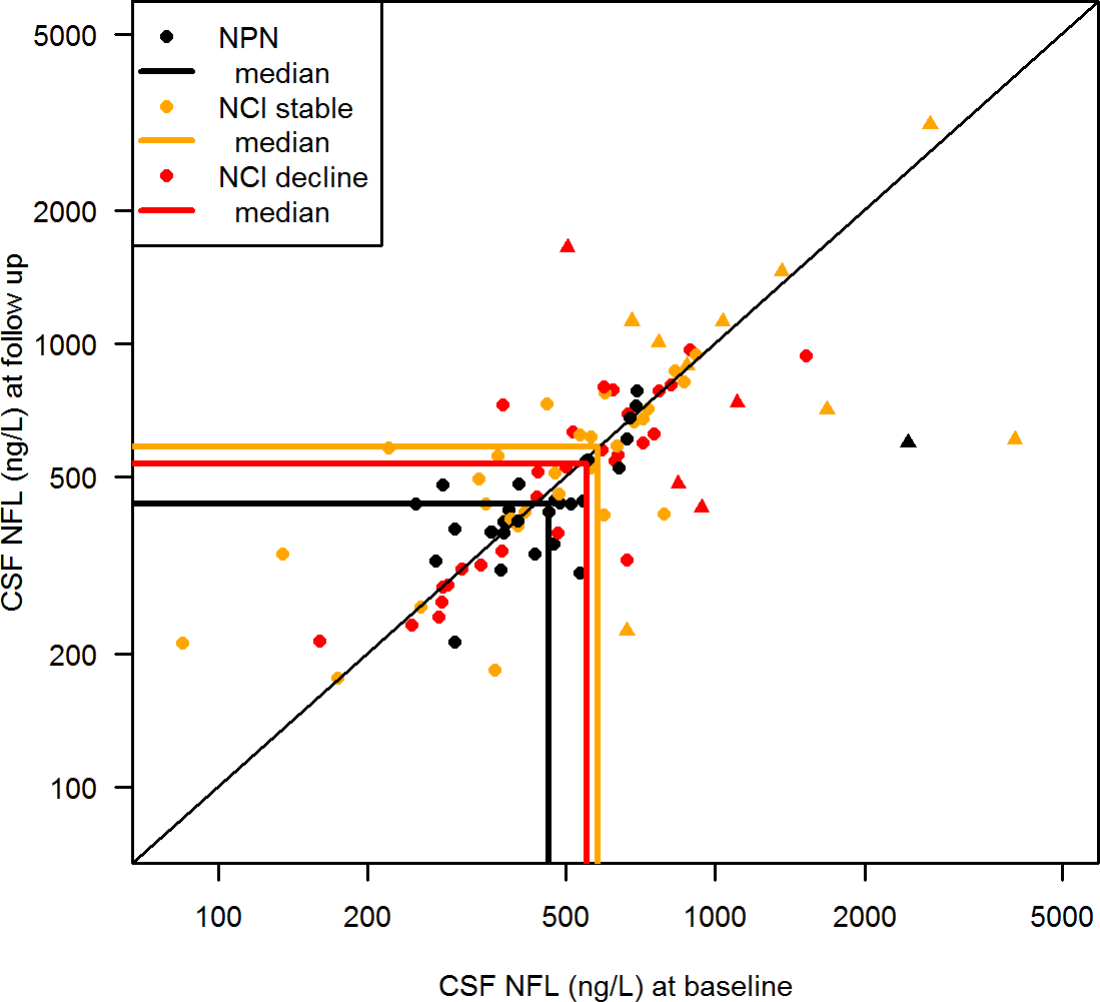
CSF NFL in relation to neurocognitive status and longitudinal stability. CSF NFL at baseline (x-axis) and follow up (y-axis) in neurocognitively normal (NCN) and neurocognitively impaired (NCI) patients (stratified by stability of neurocognitive performance (NP) as NP-stable or NP-decline). Individuals are represented by circles (NFL within age-adjusted normal limits) or triangles (abnormal age-adjusted NFL) and group medians by solid lines. Change in NFL between visits is represented by the distance from the diagonal. Geometric mean CSF NFL was non-significantly higher in neurocognitively impaired (NCI) patients, demonstrated by the difference between the medians (linear mixed effects model; p = 0.27). The proportion of above-normal (triangles) to normal (circles) CSF NFL was higher in NCI patients (19%) than in NCN patients (1%) (Fishers exact test; p = 0.06). CSF NFL in relation to neurocognitive stability was analyzed in the NCI group, where 32/70 patients had a NP-decline (red) and 38 patients were NP-stable (yellow). No difference in CSF NFL was seen between NCI stable and NCI decline subjects, illustrated by the narrow distance between the medians (linear mixed effects model; p = 0.5). Overall, there was no significant change from baseline to follow up in CSF NFL in the treatment groups, demonstrated by the symmetry around the diagonal.

Fig 2 shows the relationship between NFL and neurocognitive status in the study subjects. Although the difference corresponded to 18% higher geometric mean CSF NFL levels in the NCI group, this was not statistically significant in linear mixed effects model analysis (p = 0.12). Moreover, CSF NFL increases with normal ageing, and after adding age as a covariate in the model, the corresponding difference in CSF NFL levels between study groups was 12% (p = 0.27).

**Fig 3:**
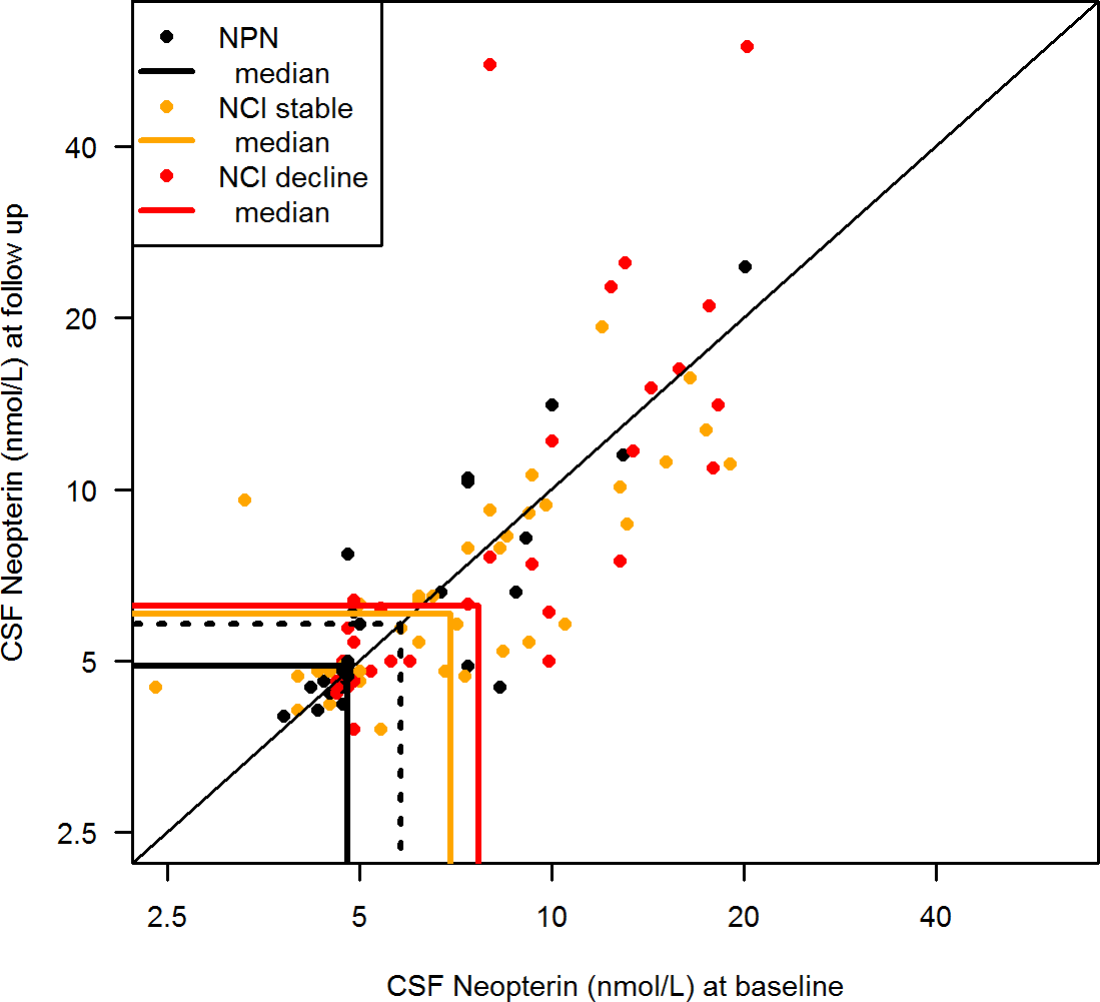
CSF neopterin in relation to neurocognitive status and longitudinal stability. CSF neopterin at baseline (x-axis) and follow up (y-axis) in neurocognitively normal (NCN) and neurocognitively impaired (NCI) patients (stratified by stability of neurocognitive performance (NP) as NP-stable or NP-decline). Individuals are represented by circles and group medians by solid lines. Change in neopterin between visits is represented by the distance from the diagonal. Geometric mean CSF neopterin was significantly higher in neurocognitively impaired (NCI) patients than in neurocognitively normal (NCN) patients, as indicated by the distance between the medians (linear mixed effects model; p = 0.036). The upper normal reference for CSF neopterin is represented by the dotted line, and the proportion of patients with CSF neopterin above the upper normal reference was significantly higher in NCI patients (66%) than in NCN patients (43%) (Fishers exact test; p = 0.04). CSF neopterin was non-significantly higher in the NCI-decline group, compared to the NCI-stable group (linear mixed effects model; p = 0.1).

CSF neopterin levels were higher in the NCI group. Baseline median (IQR) neopterin was 7.2 (4.9–10.9) nmol/l in the NCI-group, and 4.8 (4.7–7.4) nmol/l in the NCN group. At follow up, neopterin in the NCI and NCN groups was 6.3 (4.8–10.2) and 4.9 (4.6–7.2) nmol/l, respectively. In linear mixed model analysis of log_10_ CSF neopterin, this difference corresponded to 25% higher geometric mean CSF neopterin levels in the NCI group (p = 0.04) (Fig 3).

Using the previously defined age-related laboratory cut-off levels for CSF NFL, 13/70 (19%) patients in the NCI group had NFL above the upper normal age-related reference in at least one sample, compared to only one (1/29; 3%) patient in the NCN group (p = 0.06). For CSF neopterin, 46/70 (66%) in the NCI group compared to 12/28 (43%) in the NCN group had neopterin above the upper normal reference of 5.8 nmol/l on at least one occasion (p = 0.04).

CSF NFL was significantly correlated to CSF neopterin in the NCI group (r = 0.30; p = 0.0003), whereas no correlation between the biomarkers was seen in the NCN group (r = -0.13; p = 0.3). Additional weak correlations found between CSF NFL or neopterin and other factors (current or nadir CD4^+^ T-cell count, time undetectable in plasma) did not remain significant predictors of either biomarker in multivariate analysis. Although the time between visits varied in the study population, no correlation was found between time between visits and change in CSF NFL or CSF neopterin.

### CSF biomarkers and progression of impairment

Overall, 32 subjects within the NCI group experienced a NP-decline from baseline to follow up, while 38 subjects remained stable. No significant difference in CSF NFL was seen between NCI-stable or NCI-decline subjects (p = 0.5) (Fig 2). Median (IQR) CSF NFL at baseline was 580 (374–798) ng/l in NCI-stable and 555 (347–744) ng/l in NCI-decline subjects. At follow up, corresponding CSF NFL was 586 (409–786) and 536 (320–735) ng/l, respectively. Additionally, no significant difference was found in the proportion of patients with abnormal age-adjusted CSF NFL in the subjects with NCI decline (4/32) compared to NCI-stable patients (9/38) (p = 0.4)

Median (IQR) baseline CSF neopterin was 7.0 (4.9–9.4) nmol/l in NCI-stable and 7.7 (4.9–13.0) in NCI-decline subjects. Corresponding CSF neopterin at follow up was 6.1 (4.8–9.3) and 6.3 (4.9–13.6) nmol/l, respectively. Although geometric mean CSF neopterin was 21% higher in NCI-decline patients, this difference did not reach statistical significance (p = 0.1) (Fig 3). Furthermore, we found no significant difference in the proportion of abnormal CSF neopterin in patients with NCI-decline (12/32) compared to NCI-stable patients (12/38) (p = 0.6).

## Discussion

The clinical and pathological importance of the milder HAND forms (particularly ANI) has been questioned in patients responding well to ART. In the present study of effectively treated, virally suppressed HIV-1 infected patients, we demonstrate that patients with milder forms of HAND (ANI and MND) had a higher degree of intrathecal immune activation than patients without neurocognitive impairment, suggesting that even the milder forms of HAND may represent a clinically significant pathologic process within the CNS. Moreover, the correlation between CSF NFL and neopterin found in the NCI patient group, but not in the NCN group, indicates a mechanistic association between CNS inflammation, neuronal damage and neurocognitive impairment during effective ART.

In patients with HIV-associated dementia, the most severe form of HAND, HIV-1 infection in the CNS initiates a neuropathological inflammatory response known as HIV encephalitis (HIVE). HIVE is characterized by immune activation and neuronal damage that is measurable in the CSF of patients with HIV-associated dementia [6, 11, 12, 23, 24]. However, the pathogenesis of the milder HAND forms prevalent during effective antiretroviral therapy is less clear, and may differ from that of more severe HAND [25].

Previous studies have shown substantially increased levels of CSF NFL in patients with HAD, and mild elevations in some untreated neurologically asymptomatic patients, mainly with low CD4^+^ T-cell counts [11, 12]. However, CSF NFL is frequently normalized after initiation of ART, although a low level elevation within the normal range has been detected in virally suppressed patients when compared to HIV-negative healthy controls [12, 26]. In the current analysis, we did not see any statistically significant difference in age-adjusted CSF NFL between the two study groups, although the study may have been underpowered to detect a low level difference in CSF NFL. Moreover, the majority of subjects had CSF NFL within the normal range, although the proportion of patients with abnormal CSF NFL levels was higher in the NCI group with a trend seen towards significance (p = 0.06). The frequency of abnormal NFL in the NCI group was 20%, which is substantially higher than numbers reported in previous studies of virally suppressed patients [12, 27]. In the NCN group, the number of patients with CSF NFL within the normal range (97%) was comparable to numbers previously found in HIV-negative healthy controls [12]. Additionally, the correlation found between CSF NFL and neopterin in the NCI group, has previously only been reported in untreated patients but not in patients with effective ART [12]. No correlation between the biomarkers was seen in the unimpaired subject group, suggesting that neurocognitive impairment might reflect a discrete, continuous neuroinflammation and axonal degradation at least in some individuals, despite otherwise effective ART.

The higher levels of CSF neopterin found in subjects with NCI in the current analysis suggest that even the milder forms of HAND may result from an ongoing pathological process within the CNS. However, we found no clear biomarker evidence of a link between neuroinflammation and neurocognitive decline. All of the NCN subjects in the study were stable in neurocognitive performance, including only NCI subjects in the evaluation of CSF biomarkers in relation to neurocognitive decline. Although neopterin tended to be higher in subjects with baseline impairment and NP-decline, this difference was non-significant (p = 0.1). Neopterin is a sensitive marker of macrophage and microglial activation, and almost all of the neopterin detected in CSF originates from inside the CNS [28]. Although ART greatly reduces intrathecal immune activation, CSF neopterin remains elevated in a significant proportion of patients despite viral suppression [13]. Whether this residual intrathecal immune activation results from persistent low level viral replication within the CNS despite apparently effective ART or from other causes remains unclear. Using sensitive assays, patients with detectable low-level residual CSF virus had higher neopterin levels than subjects with undetectable HIV-1 RNA in CSF, indicating that elevated CSF neopterin is intimately correlated to CSF viral load [29, 30]. Although all but one subject included in the current analysis had CSF HIV-1 RNA below the detection level of 50 copies/ml in routine clinical PCR assays at baseline, we cannot rule out a difference between subject groups in low-level CSF viral load if more sensitive assays had been used, and that such differences could have a potential influence on intrathecal immune activation. Additionally, we cannot rule out that differences in ART adherence may have influenced results. Although only six subjects had detectable virus in plasma or CSF at follow up, all were in the NCI group.

The currently used diagnostic criteria of HAND have been criticized for overestimating the prevalence of impairment in patients on ART, thereby obscuring identification of the actual burden of HIV-related brain disease and that milder forms of HAND (mainly ANI) may have little clinical significance [15, 16]. The use of neuropsychological testing in relation to normative controls in the diagnosis of HAND does not differentiate between ongoing brain injury and static residual damage, such as residual sequele after injuries related to advanced disease with a low CD4^+^ T-cell count prior to initiation of ART. Although the NCI group in the present study had increased immune activation as a reflection of an active immunologic process, we did not find a significant relationship between decline in neurocognitive performance and the CSF biomarkers measured in this study, indicating that effective ART may be sufficient in limiting CNS damage in most patients. However, recent data suggest an increased risk for progression to symptomatic HAND in individuals with ANI [17]. Although not all patients in the report by Grant et al. were on effective ART, a sub analysis did not reveal different results in the virally suppressed subjects, suggesting that in some individuals, the presence of a HAND diagnosis may represent a risk of progression in neurocognitive impairment despite effective ART.

In this study, all subjects were using effective, virally suppressive combination ART, and individuals with known significant comorbid conditions that could influence either NP-test result or CSF biomarkers were excluded. However, we cannot rule out the presence of background factors such as prior drug use, abdominal obesity or other components of the metabolic syndrome[31] or other differences between the study groups (unknown or not recorded) which may have influenced the findings. Additionally, the time between study visits may not have been sufficient to detect a difference in CSF biomarkers in relation to the stability of neurocognitive performance although we did not detect any differences in biomarkers in the included subjects that had a NP decline.

## Conclusions

The findings in this study indicate that milder HAND forms may be a clinically significant reflection of an ongoing pathologic process within the CNS in some patients, where viral suppression alone is not sufficient to eliminate neuronal inflammation. The correlation found between CSF neopterin and NFL in subjects with neurocognitive impairment also indicates a mechanistic association between immune activation, neuronal damage and neurocognitive impairment during effective ART. Although standard ART is likely sufficient to prevent neurologic complications in most patients, effective control of viral replication through ART alone may not be sufficient in all cases. Methods of identifying individual patients at risk of developing, as well as progressing in HAND are needed, where the use of CSF biomarkers and neuropsychological testing will likely continue to be important tools.

## Supporting Information

S1 Data (XLSX)
